# Acute Meningitis/Encephalitis Presumably Secondary to Human Herpesvirus 6: A Case Report

**DOI:** 10.7759/cureus.89609

**Published:** 2025-08-08

**Authors:** Jeffrey Gonzalez, Rima Avellan, Claudette Barreto Lopez, Kebir Bedran, Lorena Del Pilar Bonilla

**Affiliations:** 1 Herbert Wertheim College of Medicine, Florida International University, Miami, USA; 2 Family Medicine, Baptist Health South Florida, Miami, USA; 3 Internal Medicine, Baptist Health South Florida, Miami, USA; 4 Translational Medicine, Baptist Health South Florida, Miami, USA

**Keywords:** herpes virus encephalitis, hhv-6 encephalitis, hsv encephalitis, human herpesvirus 6 (hhv-6), immunocompetent adult

## Abstract

In this report, we present the case of a 39-year-old immunocompetent female with acute meningitis/encephalitis secondary to human herpesvirus 6 (HHV-6). Her initial symptoms included fever, hallucinations, and tremors, which prompted a broad diagnostic workup for infectious and autoimmune causes of encephalopathy. Her cerebrospinal fluid (CSF) initially tested negative for viral pathogens. Subsequently, a polymerase chain reaction test confirmed the presence of HHV-6 in the CSF. The patient ultimately received antiviral therapy with ganciclovir and foscarnet, which resulted in clinical improvement. This case report highlights the importance of including HHV-6 in the diagnostic workup of encephalitis in immunocompetent individuals.

## Introduction

Human herpesvirus 6 (HHV-6) is a member of the Herpesviridae family, primarily known for causing roseola in children [1]. While HHV-6 remains latent in most individuals, it can reactivate under certain conditions, particularly in immunocompromised patients and patients under two years of age, leading to complications such as encephalitis. Such cases of HHV-6 encephalitis can include a spectrum of neurological involvement, which can include confusion, ataxia, and seizure activity [2]. However, cases of HHV-6 encephalitis in immunocompetent adults are exceedingly rare, with only a handful documented in the literature [2]. Notably, epidemiological data indicate that HHV-6 infection without encephalitis in immunocompetent patients is typically acquired in more than 90% of individuals, typically by the age of three [3].

Given the nonspecific nature of its clinical presentation, HHV-6-associated infections may pose a diagnostic challenge. Patients may present with a constellation of neurological symptoms, including altered mental status, hallucinations, seizures, and tremors, which can overlap with a broad range of infectious, autoimmune, and metabolic disorders [2]. Of these symptoms, the most common presentation in immunocompromised patients with HHV-6 encephalitis tends to be altered mental status, a universal finding among published cases in all affected patients, followed by seizures in 3/7 (43%) patients. Immunocompetent patients have differing presentations, with the most common being fever in 9/17 (53%) patients and focal neurological deficits in 8/17 (47%) patients [4]. Additionally, cases of infectious encephalitis, including HHV-6 etiologies, have general diagnostic criteria that may facilitate management. This includes the presence of an altered mental status lasting at least 24 hours or more without any other identified cause [2]. This is a major criterion that is essential for the diagnosis of this form of encephalitis. Additional criteria that can further support a diagnosis of HHV-6 encephalitis include new focal neurological findings, findings suggestive of encephalitis on neuroimaging such as mesial temporal lobe hyperintensities, and new-onset seizures [2]. Further diagnostic tools that can be used in the diagnostic pathway for HHV-6 encephalitis include cerebrospinal fluid (CSF) polymerase chain reaction (PCR) analysis for HHV-6 DNA, as well as paired serum PCR to rule out integration of HHV-6 DNA into host chromosomes to exclude false-positives as chromosome-integrated HHV-6 DNA may not be indicative of an active infection [2].

The clinical course and response to antiviral therapy of the patient presented in this case report raise important considerations regarding the management and prognosis of HHV-6 encephalitis in adults. This case highlights the need for further research into diagnostic biomarkers and treatment strategies in similar cases. Our report adds to the limited body of literature on HHV-6 encephalitis in immunocompetent adults and underscores the importance of timely recognition and management of this rare but potentially severe condition.

## Case presentation

A 39-year-old female with a history of migraine headaches, hypertension, alcohol abuse, and alcoholic pancreatitis presented with fever, headache, nausea, and tremors. The patient had no relevant past medical history of other neurological disorders, including seizures. The patient initially presented with a temperature of 100.9°F, with headaches characterized as right-sided temporal migraine headaches. Per family, she was hallucinating, febrile, confused, and exhibiting tremors at work, with the onset of these symptoms beginning on the day of the patient’s visit to the emergency department. The patient’s family reported no recent changes in diet or new exposures to infectious agents or new vaccinations. Notably, though, the patient was reported to have stopped drinking alcohol three months prior, although relapse was suspected.

On initial assessment, vitals were within the reference ranges: heart rate, 86 beats/minute; respiratory rate, 18 breaths/minute; blood pressure (BP), 158/115 mmHg; and SpO_2_, 100%. Pertinent findings on physical examination included a presenting Glasgow Coma Scale score of 11 with confusion. The patient was alert to person and place but not to the situation. The patient was actively hallucinating at the time of presentation. The patient did not have slurred speech and did not present with focal neurological deficits. The rest of the physical examination, including cardiovascular, respiratory, and gastrointestinal systems, was unremarkable. At this time, possible items in the differential included viral encephalitis, viral/bacterial meningoencephalitis, alcohol withdrawal/delirium tremens, metabolic encephalopathy, and autoimmune encephalitis. Initial labs, including biochemistry, were normal except for noted hypokalemia (K⁺: 3.2), elevated aspartate aminotransferase (96 U/L), and lactic acid of 2.6 mmol/L (Table 1). Urinalysis revealed pyuria, hematuria, and moderate bacteriuria. Imaging (chest X-ray, CT of the head/abdomen) was unremarkable.

**Table 1:** Intake biochemistry findings. CBC = complete blood count; WBC = white blood cell; RBC = red blood cell; ALT = alanine aminotransferase; AST = aspartate aminotransferase; BUN = blood urea nitrogen; Cr = creatinine

Biochemistry	Reference range	Day 1
CBC with differential		
WBC (×10³/µL)	3.40–11.0	4.82
RBC (×10⁶/µL)	3.80–5.20	3.67 (L)
Hemoglobin (g/dL)	12.0–15.0	10.5 (L)
Hematocrit (%)	35.0–45.0	30.4 (L)
Platelets (×10³/µL)	130–360	316
Metabolic panel		
Sodium (mEq/L)	136–145	135 (L)
Potassium (mEq/L)	3.5–5.1	3.2 (L)
Chloride (mEq/L)	98–107	93 (L)
Bicarbonate (mEq/L)	21–32	29
Calcium (mg/dL)	8.5–10.1	9.6
Glucose (mg/dL)	70–126	137 (H)
Liver function		
ALT (U/L)	16–65	19
AST (U/L)	8–37	96 (H)
Total bilirubin (mg/dL)	0.2–1.0	0.3
Kidney function		
Creatinine (mg/dL)	0.55–1.02	0.78
BUN (mg/dL)	7–18	8
BUN/Cr ratio	12.0–20.0	10.3 (L)
Lactic acid	0.5–2.2 mmol/L	2.6 mmol/L (H)

In the absence of signs of acute stroke or cardiac ischemia, encephalitis of unknown etiology was considered the leading differential diagnosis at the time due to the presence of encephalopathic features, including confusion, hallucinations, and tremors. Initial empiric therapy included bacterial coverage of ceftriaxone 2 g IV every 12 hours and vancomycin 15 mg/kg IV every 12 hours. Viral coverage was also done with acyclovir 10 mg/kg IV every eight hours. The patient was also administered a bolus of 1 L of normal saline and lorazepam 1 mg IV for agitation and hallucinations. Treatment was also started to treat the patient’s hypertensive urgency, which was suspected of being secondary to autonomic dysregulation in the setting of encephalopathy and/or alcohol withdrawal. The patient was provided with hydralazine 10 mg IV once, which provided a BP reduction to ~140/90 mmHg within two hours. Despite treatment, the patient remained confused with hallucinations and tremors. She was admitted for altered mental status, possible alcohol withdrawal, and hypertensive urgency without evidence of end-organ damage. Due to worsening encephalopathy, she was transferred to a progressive critical care step-down unit and started on a nicardipine drip at 5 mg/hour IV, after which the patient’s BP remained at a stable ~140/90 mmHg.

On day two, Infectious Disease was consulted due to the initial concerns for a viral/bacterial meningencephalitis and recommended to continue empiric antibiotics. The service also ordered a CSF analysis as the patient had been stabilized, and initial brain CT imaging had ruled out mass effect. Blood cultures at this time continued to exhibit no growth, and an echocardiogram revealed mild concentric left ventricular hypertrophy, normal ejection fraction (65-70%), and no vegetations. Brain MRI showed findings concerning for meningitis, but no ischemia or hemorrhage; there was a possible CSF artifact from motion.

On day three, lumbar puncture results (Table 2) indicated a potential viral etiology with neutrophilic response in the light of a negative Gram stain and ongoing negativity. Additionally, further CSF analysis was performed with a meningencephalitis panel to attempt to localize the specific etiology of the patient’s encephalitis. This panel tested for a variety of possible sources, including viral and bacterial (Table 2). Furthermore, an autoimmune workup was also conducted, which was ultimately largely unremarkable, with only a positive value in lupus antibody testing. Due to a largely still unknown definite etiology for the patient’s encephalopathic presentation, in addition to negative bacterial cultures, CSF pleocytosis, and a positive lupus antibody panel, neurology was consulted and recommended starting the patient on steroid treatment for the possibility of autoimmune encephalitis. The patient was placed on methylprednisolone 1 g IV daily for five days.

**Table 2:** CSF analysis and autoimmune workup day three. CSF = cerebrospinal fluid; PCR = polymerase chain reaction; CRP = C-reactive protein

CSF analysis			Autoimmune workup		
Day	Test	Result	Reference range	Test	Result
Day 3	Total nucleated cells	131 (0–5 cells/µL)	<14 IU/mL	Rheumatoid factor	Negative
	Protein	225 (15–45 mg/dL)	Negative	Antinuclear antibody	Negative
	Glucose	63 (40–70 mg/dL)	<0.9 U/mL	Lupus/ENABS RNP antibody	7.3
	Meningoencephalitis panel (PCR)	Negative	Negative	NMDAR antibody	Negative
		-	<1.0 mg/dL	CRP	58.7

On day five, electroencephalography showed mild diffuse encephalopathy without seizure activity. On day six, a repeat lumbar puncture showed elevated protein and glucose; the meningococcal panel returned positive for HHV-6 and no other pathogenic sources, confirming a possible HHV-6 meningococcal encephalitis (Table 3). Additionally, fungal and bacterial cultures also returned negative at the time, with no growth detected. At the time, this was a likely HHV-6 reactivation due to the high frequency of HHV-6 infections in childhood. At this point, chromosome-integrated HHV-6 was not yet ruled out.

**Table 3:** CSF analysis on day six. CSF = cerebrospinal fluid

CSF analysis		
Differential		
Day	Test	Result
Day 6	Total nucleated cells	131 (0–5 cells/µL)
	Protein	156 (15–45 mg/dL)
	Glucose	115 (40–70 mg/dL)
	Red blood cells	14 (0–5 cells/µL)
Meningoencephalitis panel		
HHV-6	Detected	
Cytomegalovirus	Not detected	
Varicella zoster Virus	Not detected	
Enterovirus	Not detected	
Parechovirus	Not detected	
Neisseria meningitidis	Not detected	
Streptococcus pneumoniae	Not detected	
Haemophilus influenzae	Not detected	
Listeria monocytogenes	Not detected	
Cultures		
Fungal	No growth	
Bacterial	No growth	

**Figure 1:**
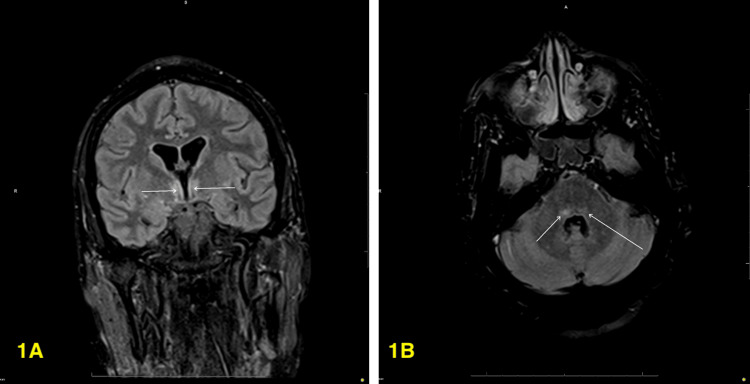
Brain fluid-attenuated inversion recovery (FLAIR) MRI. (A) Coronal T2/FLAIR images demonstrate an abnormal, symmetric hyperintense signal involving the columns of the fornices. (B) Axial T2/FLAIR images demonstrate an abnormal hyperintense signal involving the right greater than left dorsal pons.

On day 14, repeat MRI supported findings of encephalitis (Figures 1A, 1B). Despite the positive lupus antibody, the CSF autoimmune panel was negative. Multidisciplinary consensus (Neurology, Internal Medicine, Intensive Care Unit) favored HHV-6 encephalitis over autoimmune etiology. She was treated with ganciclovir (days 7-24) and foscarnet (days 14-26), with significant improvement in the neurologic examination thereafter. The significant improvement after treatment with both medications allowed the multidisciplinary team to rule out chromosome-integrated HHV-6 and focus on a primary diagnosis of HHV-6 encephalitis in the setting of a reactivation.

Her hospital course was complicated by aspiration pneumonia (treated with six days of meropenem), intubation (extubated day 18), and prerenal acute kidney injury due to spironolactone, angiotensin receptor blockers, and foscarnet. Urine analysis showed granular casts consistent with acute tubular necrosis. She also developed anemia requiring packed red blood cell transfusion. Hypothermia prompted endocrine evaluation, but cortisol and adrenocorticotropic hormone stimulation tests were within reference ranges.

She was stabilized with improvement of her neurological examination and discharged to acute rehabilitation on day 43 with a modified Rankin Scale score of 3, indicating moderate disability. The patient continued to have difficulties walking on discharge. The patient was to follow up with Neurology and Infectious Disease one week after discharge.

## Discussion

HHV-6 encephalitis in adults typically presents in immunocompromised individuals, such as those under treatment for transplant or immunosuppressant medication. However, reports of HHV-6 encephalitis in immunocompetent individuals are rare, with fewer than 15 documented cases in English-language, peer-reviewed literature. Typical HHV-6 encephalitis, which is more commonly observed in younger and/or immunocompromised patients, involves a constellation of symptoms, including seizures, altered states of consciousness, ataxia, and other significant neurological deficits [2]. Previously reported cases of HHV-6 encephalitis have involved symptoms similar to what our patient experienced, including progressively worsening altered mental status from symptom onset [2]. Our patient, on presentation, did exhibit some of the above-mentioned more commonly observed symptoms of HHV-6 encephalitis, which included fever and nonspecific neurological symptoms of headache and nausea, as well as altered mental status, not being alert to person, time, or place. The patient, however, did not present with signs of focal neurological deficits. In comparing these symptoms, the patient presented with classical signs of HHV-6 encephalitis, although these are non-specific and require further assessment to narrow down their etiology.

Additionally, laboratory assessment of the patient’s CSF revealed pleocytosis with a total nucleated cell count of 131 cells/µL. This was comparable to prior cases of adult-onset, PCR-positive HHV-6, which had pleocytosis within the CSF in a range of 9-155 cells/µL [4]. While our patient, like prior reported HHV-6 adult cases, exhibited symptoms of encephalitis, it must be considered that the nature of HHV-6 integration into host chromosomes can make the actual diagnosis challenging. By extension, just because patients may have a positive CSF sample for HHV-6 does not mean that they have the associated encephalitis or infection. Furthermore, our patient was tested for the presence of HHV-6 DNA in CSF, but specific titers were not obtained. While this is a limitation in conclusively determining HHV-6 infection, it should be factored that, often, the HHV-6 encephalitis diagnosis must be reached through a combination of CSF analysis and clinical context.

This is why most diagnosed cases typically involve younger patients who are more susceptible to HHV-6 infection [5]. On the other hand, there can be more ambiguity in the diagnosis of HHV-6 encephalitis in adult patients, as such cases can present with HHV-6 DNA in the CSF that could be indicative of either active infection or chromosomal integration secondary to prior exposure. This complicates diagnostic measures in patients with symptoms suggestive of this etiology [6,7]. Furthermore, the patient’s follow-up MRI indicated areas of symmetric hyperintense signals in the fornices and the pons (Figures 1A, 1B). These findings, particularly the affected fornices, are consistent with memory deficits and diminished white matter integrity, indicating potential involvement between herpesviruses and encephalitis, as in the case of herpes simplex encephalitis [8]. Furthermore, HHV-6 encephalitis has been observed affecting the mesial temporal lobes and limbic system structures, which include the fornices. This correlates with the hyperintensities observed in the fluid-attenuated inversion recovery images (Figure 1A), presenting compelling imaging findings for HHV-6 encephalitis [9]. Further expanding on a prior point, while not directly observed in our patient, HHV-6 encephalitis may also present with T2-weighted imaging brain MRI temporal lobe hyperintensities [2]. These neuroimaging findings are essential in identifying the extent of the impact of HHV-6 encephalitis; however, presentations may vary, and limited data are available to establish a conclusive pattern for HHV-6 encephalitis due to the rarity of these cases.

Potential clues indicative of active infection of HHV-6 leading to encephalitis can include responsiveness to ganciclovir, which has been reported in prior immunocompetent patient cases with HHV-6-positive CSF [10-12]. Upon potential diagnosis of this patient with HHV-6 encephalitis, prompt administration of ganciclovir in addition to foscarnet produced sustained clinical improvement. This highlights the potential efficacy of ganciclovir use in cases of immunocompetent, adult-onset HHV-6 encephalitis. Additionally, it should be noted that while the aforementioned treatment methodology produced sustained clinical improvement, spontaneous resolution from herpesviruses can occur [3], and this is also not a controlled comparison. It is more indicative of correlation, but not enough data are available to prove causation of symptoms is secondary to HHV-6.

While the patient ultimately recovered, questions have been raised regarding the potential long-term complications of HHV-6 infection. While there is no current notable literature discussing long-term outcomes of HHV-6 encephalitis in adult, immunocompetent patients, some noted outcomes have been reported for immunocompromised patients. Such patients who have experienced HHV-6 encephalitis, including those who have undergone organ transplantation, often continue to experience neurological sequelae, including impairment of memory and anterograde amnesia [13,14]. Furthermore, the ongoing long-term complications of HHV-6 encephalitis occur largely due to other neurological complications after the initial insult by HHV-6 to the CNS, not the encephalitis itself [13,14].

A notable topic regarding this is dementia, particularly Alzheimer’s disease (AD). A review of several studies found statistically significant increased presence of HHV-6 DNA in AD-positive brain specimens compared to non-AD controls [15]. Additionally, the etiology of AD, which is rooted in AB peptide fibrillization, has been associated with HHV-6, indicating that glycoproteins in herpesviruses may bind to the amyloid fibrils and promote their deposition in brain tissue [16]. Though the association is not strong enough to provide a causal relationship, it indicates a potential contributory relationship between HHV-6 and dementia development [15,16]. While possible associations between HHV-6 and dementia have been assessed, the extent of these associations must be further studied to determine the effect, if any, of HHV-6.

## Conclusions

Ultimately, HHV-6 infection, while uncommon in adults, can lead to possible HHV-6 encephalitis cases, as seen in our patient. This case highlights the importance of including HHV-6 as a possible pathogenic cause of encephalitis, even in immunocompetent individuals. Our patient exhibited common signs of HHV-6 encephalitis, including headaches and altered mental status. Additionally, clinical presentations must be paired with neuroimaging studies, which can more commonly affect the medial temporal lobes, although this was not a finding in our patient. While the exact mechanism for this is unclear, understanding the existence of such infections can allow for the development of treatment protocols while also studying the long-term implications in immunocompetent adults. While there is currently insufficient data for this demographic, further studies and assessments could go a long way in the development of guidelines for such infections and further encourage research.
